# Quantitative measures of corneal transparency, derived from objective analysis of depth-resolved corneal images, demonstrated with full-field optical coherence tomographic microscopy

**DOI:** 10.1371/journal.pone.0221707

**Published:** 2019-08-28

**Authors:** Romain Bocheux, Pascal Pernot, Vincent Borderie, Karsten Plamann, Kristina Irsch

**Affiliations:** 1 Laboratoire d'Optique et Biosciences (LOB)–École polytechnique, CNRS UMR 7645, INSERM U 1182, Institut polytechnique de Paris, and LOA–ENSTA ParisTech, École polytechnique, CNRS UMR 7639, Institut polytechnique de Paris, Palaiseau, France; 2 Vision Institute / Quinze-Vingts National Eye Hospital / GRC32 / CIC1423 –Sorbonne University, CNRS UMR 7210, INSERM U 968, Paris, France; 3 Laboratoire de Chimie Physique–Université Paris-Sud, CNRS UMR 8000, Orsay, France; Nicolaus Copernicus University, POLAND

## Abstract

Loss of corneal transparency, as occurs with various pathologies, infections, immune reactions, trauma, aging, and surgery, is a major cause of visual handicap worldwide. However, current means to assess corneal transparency are extremely limited and clinical and eye-bank practice usually involve a subjective and qualitative observation of opacities, sometimes with comparison against an arbitrary grading scale, by means of slit-lamp biomicroscopy. Here, we describe a novel objective optical data analysis-based method that enables quantifiable and standardized characterization of corneal transparency from depth-resolved corneal images, addressing the demand for such a means in both the laboratory and clinical ophthalmology setting. Our approach is based on a mathematical analysis of the acquired optical data with respect to the light attenuation from scattering processes in the corneal stroma. Applicable to any depth-resolved corneal imaging modality, it has been validated by means of full-field optical coherence tomographic microscopy (FF-OCT or FF-OCM). Specifically, our results on *ex-vivo* corneal specimens illustrate that 1) in homogeneous tissues, characterized by an exponential light attenuation with stromal depth (*z*), the computation of the scattering mean-free path (*l*_*s*_) from the rate of exponential decay allows quantification of the degree of transparency; 2) in heterogeneous tissues, identified by significant deviations from the normal exponential *z* -profile, a measure of exponential-decay model inadequacy (e.g., by computation of the Birge ratio) allows the estimation of severity of stromal heterogeneity, and the associated depth-dependent variations around the average *l*_*s*_ enables precise localization of the pathology.

## Introduction

Vision is dependent upon the transparency of the cornea, which largely relates directly to stromal structure, more precisely the regular microstructure of the lamellae and the nanostructure of the closely packed collagen fibrils that are embedded in an optically homogeneous ground substance [1,2]. Corneal transparency can be compromised by various pathologies, infections, immune reactions, trauma, aging, and surgery, all of which result in increased light scattering. In some cases, surgical intervention, involving the replacement of the affected cornea by donated tissue (the so-called graft that is taken from a recently deceased individual), becomes the only treatment option.

Over 10 million people worldwide suffer severe visual handicap due to loss of corneal transparency. Of these, over 1.5 million go blind every year, often due to poor suitability for corneal transplantation, or due to a shortage of available grafts [3]. For those in whom grafting may be possible, success rates range from 60% to 90% [4].

Despite its significance, means to assess corneal transparency are extremely limited. In current clinical practice, corneal transparency evaluation usually involves a gross observation of opacities using a slit-lamp biomicroscope, sometimes with comparison against an arbitrary and subjective grading scale (from 0 to 4 or 5) [5,6]. Similarly, aside from an excellent endothelial quality control of corneal grafts by means of specular microscopy, stromal quality assessment is challenging in eye banks, and generally limited to undetailed slit-lamp and/or light microscope examination. Due to the subjective and qualitative nature of the examination, results are also observer-dependent, difficult to standardize, and lack reproducibility.

Several attempts have been made to quantify and/or objectively assess corneal transparency [7], including via slit-lamp biomicroscopy [7,8], the Scheimpflug principle [9], confocal microscopy [10], and optical coherence tomography (OCT) [11], each one having its own advantages and disadvantages. However, none of these approaches have been found suitable to gain widespread usage. There is, thus, a material need for reliable and easy-to-use clinical tools and diagnostic procedures for objective and quantitative characterization, including monitoring ability, of corneal transparency, towards effective prevention, diagnosis, and treatment of various pathologies.

In this paper, we describe a novel approach that addresses this unmet need in ophthalmology by deriving quantitative measures of corneal transparency, such as the scattering mean-free path and tissular heterogeneity, from objective analysis of depth-resolved corneal images. Applicable to any depth-resolved corneal imaging modality, we demonstrate the feasibility of our optical data analysis-based method by means of full-field optical coherence tomographic microscopy (FF-OCT or FF-OCM).

## Materials and methods

### Instrumentation, image acquisition and pre-processing

**Fig 1:**
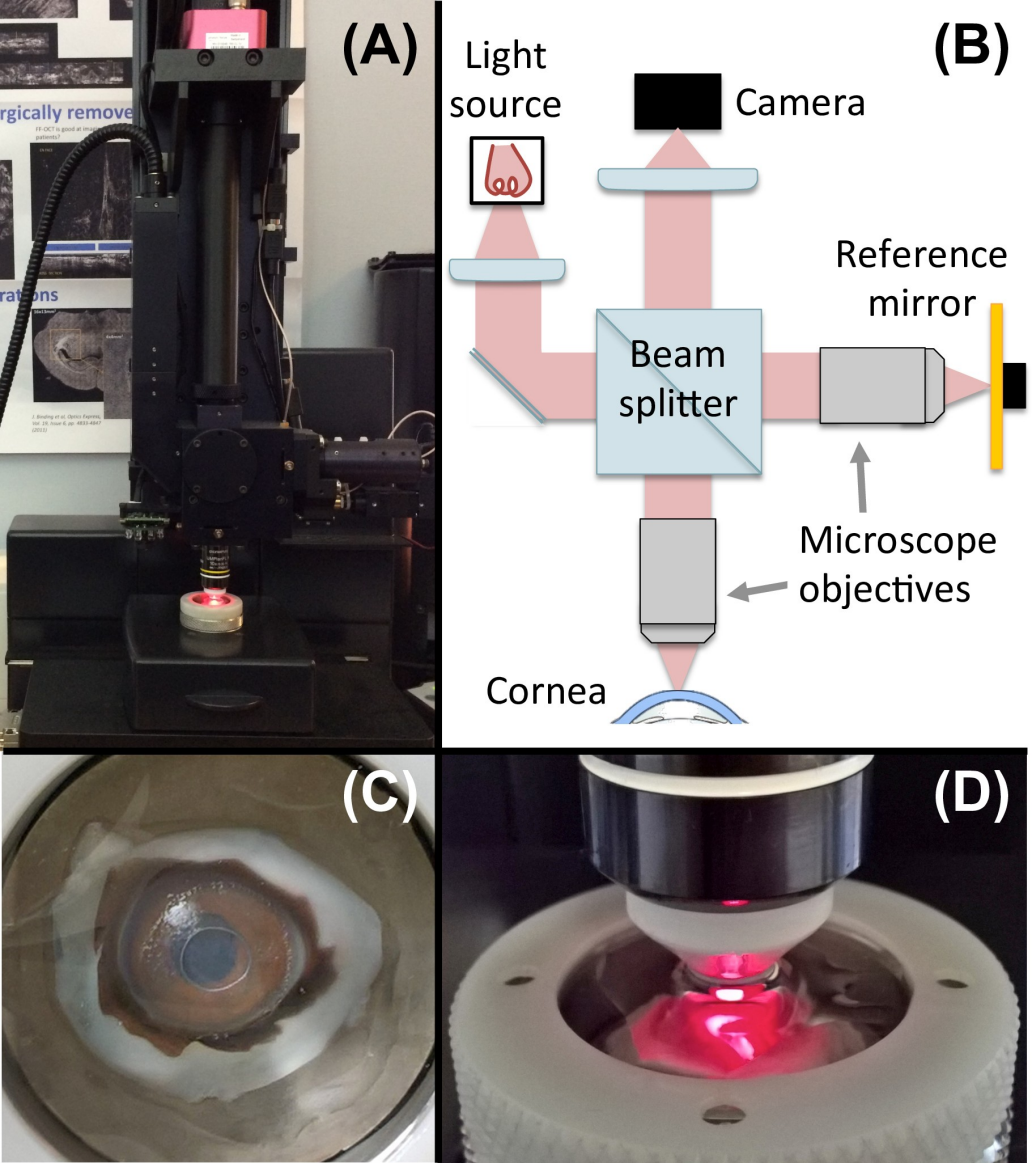
Schematic and photographs of the FF-OCT system used in this study. (A) Photograph of the commercial FF-OCT device (Light-CT, LLTech, Paris, France). (B) Schematic and optical setup of the device. (C) Photograph of corneal tissue in the sample holder. (D) Zoom on the immersion objective and the sample holder.

In this study, volumetric corneal images were acquired with a commercial FF-OCT device (Light-CT, LLTech, Paris, France; Fig 1), which has been described in detail previously [12–14]. Briefly, developed for *ex-vivo* microscopy on stationary tissue samples, this imaging modality combines elements of both OCT [15–17] and confocal microscopy [18] thereby providing cross-sectional and *en-face* views at high resolution. More specifically, the FF-OCT system uses a Halogen lamp as spatially incoherent light source in a Linnik-based interferometer configuration with high-numerical aperture immersion objectives and extracts the signal from the background of incoherently backscattered light using a phase-shifting method. *En-face* 2D tomographic images are acquired without lateral scanning; by scanning in the axial direction, in-depth 3D structural information of biological tissues, including the cornea [14,19], at cellular resolution can be visualized.

Acquired corneal images consisted of 1024 x 1024 pixels over a 780 x 780 μm field of view with 1.6-μm transverse x 1.0-μm axial resolutions. Image acquisition time for a typical 500-μm-depth stack in the cornea, acquired every micron in depth, is under five minutes. Throughout image acquisition, the cornea remained immersed in a closed chamber filled with storage medium, decreasing any potential risk of contamination [19].

FF-OCT images were pre-processed and analyzed offline using custom software developed in Matlab (Mathworks, Inc., Natick, MA, USA). Pre-processing included 3D-image segmentation and flattening of the corneal surface. Briefly, for each row of the 3D-image stack, that is, for each 2D cross-sectional image, the position of the corneal surface was determined by finding the local maximum (around the epithelium) using a thresholding procedure and smoothing the obtained data by applying a median filter as well as a Savitsky-Golay filter (i.e., a convolutional low-order polynomial filter); image flattening was then performed by shifting the 3D-data set according to the smoothened surface positions.

### Image analysis-based approach and fitting algorithm

**Fig 2:**
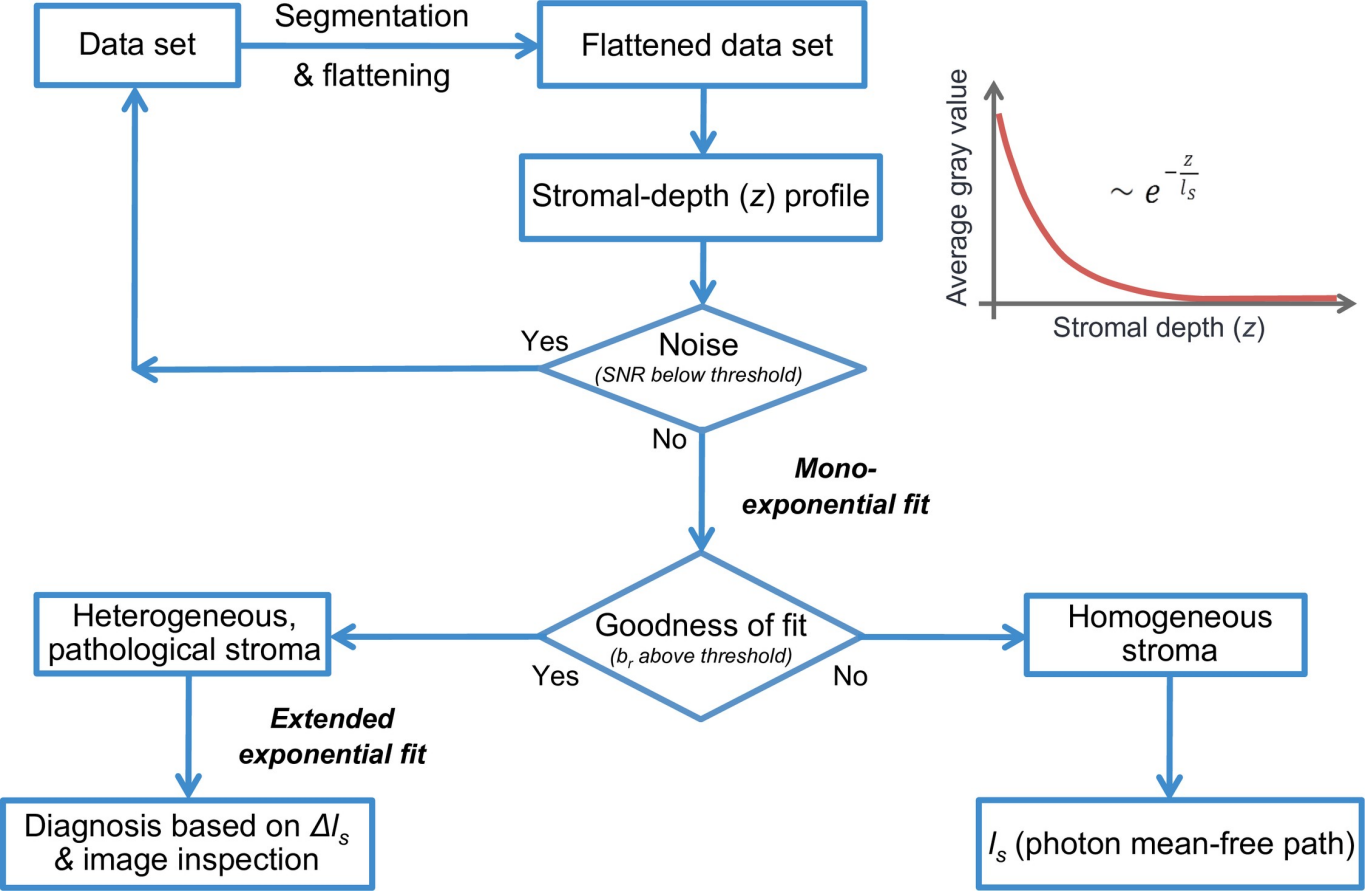
Flowchart and summary of our objective image-analysis based approach to corneal transparency quantification. From the FF-OCT data set, after segmentation and flattening of the corneal surface, we extract the stroma and generate mean amplitude depth profiles by computing the average grey value over the entire field of view for each *en face* image in depth. Next, the random noise component is estimated as the residuals of a smoothing spline function fit, from which the signal-to-noise ratio (SNR) is calculated. If the SNR is above a certain threshold, we proceed with the mono-exponential fitting procedure and estimate the goodness of the fit by means of the Birge ratio (*b*_*r*_) or reduced chi-square statistic. If the Birge ratio is below a certain threshold, the exponential decay model is considered satisfactory, and we regard the stroma as homogeneous and quantify the degree of transparency by calculating the photon mean-free path (*l*_*s*_) from the rate of amplitude decay. If the Birge ratio is above a certain threshold, the decay model may not be an adequate representation of the data, and we understand that the stroma is heterogeneous with pathologies and proceed with the estimation of the parameters of an extended decay model, with variable, depth-dependent *l*_*s*_, using a Bayesian approach. From the estimated deviations from the average *l*_*s*_ value (*Δl*_*s*_) along stromal depth, one can then localize the pathologies and confirm the diagnosis by image inspection.

Our approach (illustrated in Fig 2) is based on a mathematical analysis of the acquired optical data as a function of depth in the cornea. Specifically, after 3D-image pre-processing, as described previously, and extraction of the corneal stroma, the data is analyzed with respect to the propagation properties of the coherent mean of backscattered light in depth of the corneal volume.

Loss of corneal transparency is reflected in an increase of light scattering induced by loss of short-range order of stromal collagen fibrils [2]. Since the targeted application is the early detection of transparency loss, single scattering will be dominant, and it may be shown that the average of “ballistic photons” or the coherent mean of backscattered light is attenuated (along stromal depth, *z*) by scattering processes following a Lambert-Beer law. The *z*-dependent FF-OCT signal may thus be written as:

$$A(z)=A_{0}\exp(-\mathrm{Bz})+C$$ (1)

with an amplitude factor *A*_*0*_ (note that FF-OCT measures the amplitude of the light backscattered by the sample rather than its intensity) associated with the directionality of the scattering process and an additive constant *C* indicative of the baseline or background of multiply-scattered photons. The scattering or photon mean-free path, *l*_s_ (a major indicator of scattering extent and thus of transparency of a medium) can be calculated by investigating the exponential attenuation of the signal (*l*_s_ = 1/*B*). Any significant deviation from such an exponential decay (or a deviation from a linear logarithmic regression [20]), that is a deviation beyond a certain threshold, on the other hand, is expected to indicate the presence of pathology, the severity of which may be estimated by the overall goodness of the fit.

To differentiate the inadequacy of a mono-exponential model (due to presence of a pathology) from mere measurement noise, we proceed in three steps:

1. Estimation of the random noise component as the residuals of a smoothing spline function;
2. Bayesian inference of the parameters of a mono-exponential decay model and comparison of the fit residuals to the random noise; in case of significant model inadequacy,
3. Bayesian inference of the parameters of an *extended decay model*, with variable, *z*- dependent *l*_*s*_.

The Bayesian models are implemented in stan [21], using the *rstan* interface package for *R* (*R* Core Team, Vienna, Austria). The main functions are available as an R package at https://doi.org/10.5281/zenodo.1494972, as are graphical and script-based interfaces, along with a detailed vignette of the code implementation, at https://doi.org/10.5281/zenodo.1494973.

#### Estimation and modeling of random noise

A cubic smoothing spline function is fitted to the depth-dependent data to estimate its random component. That is, the residuals of the smoothing spline fit are assigned to measurement noise of the stromal-depth profile, from which the signal-to-noise ratio (SNR) is computed (using the respective averages). The noise distribution is then fitted with a normal distribution of zero mean and a standard deviation following an exponential decay, using Bayesian inference [22]. This noise model is used in the following steps.

#### Estimation of mono-exponential decay model adequacy

The parameters of the mono-exponential decay (Eq 1) are estimated by Bayesian inference, with a Gaussian likelihood based on the sum of squared residuals, weighted by the noise model. The quality of the mono-exponential fit is estimated by inspection of the residuals, conforming with the random experimental noise, by means of the Birge ratio (*b*_*r*_, the reduced weighted chi-squared). If *b*_*r*_ is below a certain threshold, the mono-exponential decay model is considered an adequate representation of the data, and we proceed with the extraction of the fit parameters from the data, including the scattering mean-free path, *l*_*s*_, as the global parameter or quantitative measure of transparency. Otherwise, the mono-exponential decay model may not be appropriate and we perform a fit by an extended exponential decay, as follows.

#### Calibration of an extended decay model

If *b*_*r*_ is above a certain threshold, we estimate the parameters of an extended decay model, which allows the scattering mean-free path to be variable with stromal depth, according to the function:

$$A(z)=A_{0}\exp(-z/\left(l_{s}(1+{\Delta l}_{s})\right))+C$$ (2)

where Δ*l*_*s*_ represents the relative variation around the mean value of *l*_*s*_. This correction function is modeled by a Gaussian process, used as an interpolator between a set of control points on a regular grid along *z*. The values of the control points are inferred simultaneously with the other decay parameters, with degeneracy-avoiding constraints between *l*_*s*_ and Δ*l*_*s*_.

### Human corneas

Corneal images of pathological surgical specimens and healthy donor grafts were included in this study, which was approved by the Institutional Review Board (Patient Protection Committee, Ile-de-France V) and adhered to the tenets of the Declaration of Helsinki as well as to international ethical requirements for human tissues. The surgical specimens of diseased corneas were obtained from the Quinze-Vingts National Eye Hospital operating room at the time of keratoplasty. The ethics committee waived the requirement for informed written consent of patients; however, all patients provided informed oral consent to have their specimens used in research. The human donor specimens were obtained from the tissue bank of the “Établissement Français du Sang” after they were discarded before transplantation because of low endothelial cell count, according to the standards of the EU Eye Bank Association. None of the donor specimens were from a vulnerable population and all donors or next of kin provided written informed consent that was freely given. All corneal specimens were stored in a specific medium containing Dextran (CorneaJet, Eurobio, France) for deturgescence, prior to FF-OCT imaging [19].

## Results and discussion

**Fig 3:**
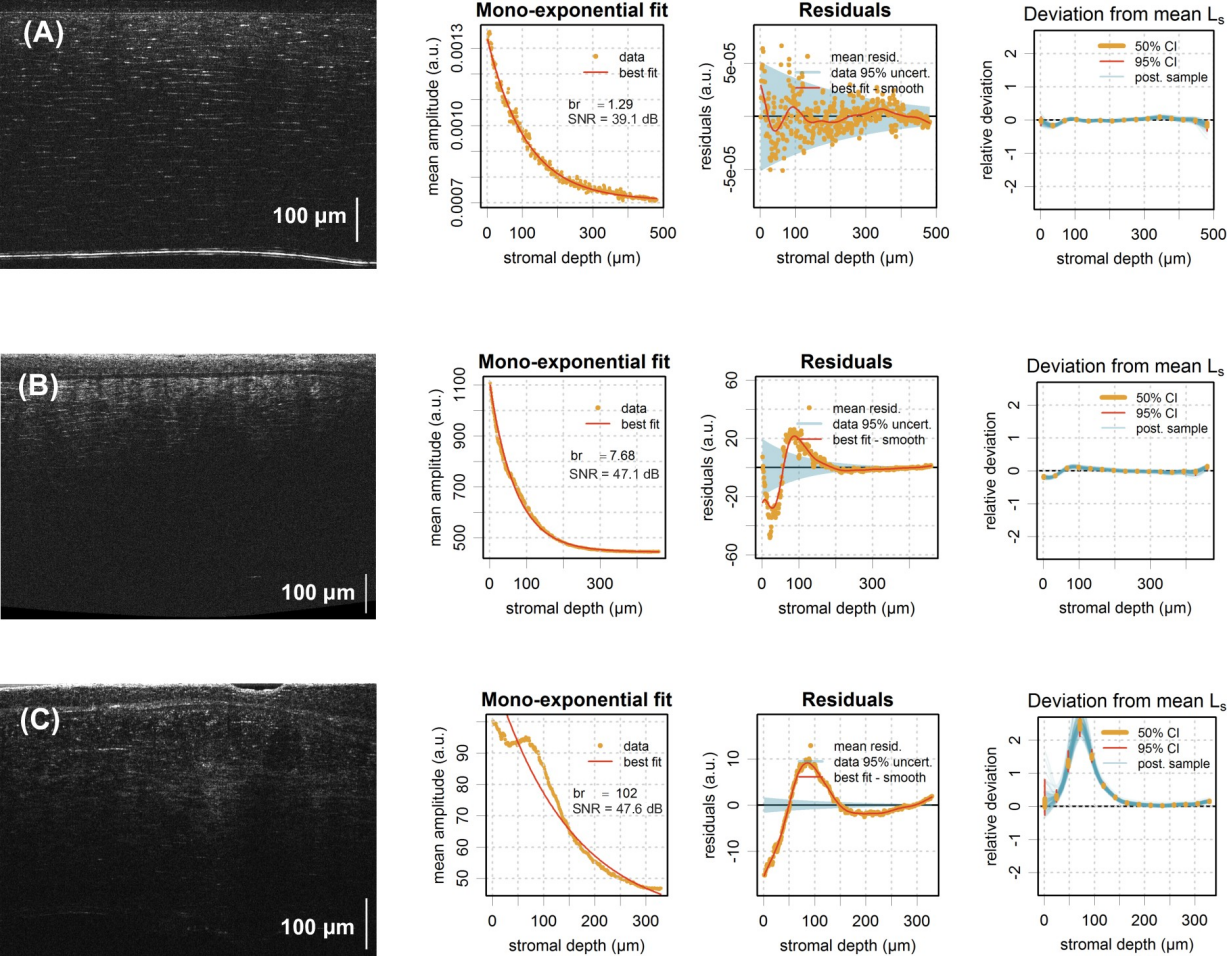
Graphical representation of results. FF-OCT cross-sectional images of typical human corneal tissues with corresponding mean amplitude depth profiles and mono-exponential fitting analysis. (A) Eye-bank (donor) cornea with intact transparency showing a normal exponential stromal depth profile, confirmed by a Birge ratio (*b*_*r*_) close to 1. (B) Pathological cornea with compromised transparency showing a faster decaying stromal depth profile and larger Birge ratio (*b*_*r*_ ~ 8), but still a weak deviation from the mono-exponential model (*b*_*r*_ ~ 8, |Δ*l*_*s*_| < 0.1). (C) Pathological cornea with heterogeneous stroma (scarring) showing a non-exponential stromal depth profile, confirmed by a Birge ratio >> 1 and a significant deviation from the average value of *l*_*s*_ (i.e., the scattering mean-free path) below 150 μm (*b*_*r*_ ~ 102, max(|Δ*l*_*s*_|) ~ 2.5).

Typical 2D cross-sectional slices through acquired 3D FF-OCT image stacks are shown in Fig 3, comprising one eye-bank (donor) cornea with intact transparency (Fig 3A), one pathological cornea with compromised transparency (Fig 3B), and one pathological cornea featuring stromal heterogeneities (scarring; Fig 3C), as per “gold-standard” subjective and qualitative image inspection.

As expected in any homogeneous scattering medium, the backscattered signal continuously decreases with stromal depth in Fig 3A and 3B. This is not the case in Fig 3C, where the presence of stromal scars is giving rise to regions of increased light backscatter (often referred to as “hyperreflective” elements).

To quantify this behavior, we generated mean amplitude stromal depth profiles, representing the grey value averaged over the entire field of view of successive *en-face* FF-OCT slices as a function of stromal depth, fitted to a mono-exponential function (second column of Fig 3).

In a first step, the random noise is estimated as described above and represented by the shaded area in the “Residuals” sub-plot (third column of Fig 3). In the first case (Fig 3A), the mono-exponential fit gives residuals that do not stray significantly from the random noise. This is confirmed by a Birge ratio close to 1 (*b*_*r*_ = 1.29). Similarly, the best fit obtained by Bayesian calibration of an extended decay is not showing any significant deviation from a mono exponential. This can be appreciated from the last column, where 50% and 95% probability intervals for the control points of the Gaussian process (vertical bars) and samples of the estimated mean-free path deviations, Δ*l*_*s*_, are shown. In the second case (Fig 3B), the residuals of the mono-exponential fit exceed the noise (*b*_*r*_ = 7.68), which statistically speaking may be considered a significant deviation and as such constitute mono-exponential model inadequacy. However, the correction term in the extended model has a weak amplitude (|Δ*l*_*s*_| < 0.1) and we therefore consider the deviation from the mono-exponential as secondary and the stroma as homogeneous.

For such homogeneous tissues, the scattering mean-free path, *l*_*s*_, may thus be used to further quantify the degree of transparency. Example results for our representative normal and pathological cases (Fig 3A and 3B) are listed in Table 1, confirming the compromised transparency, with lower mean-free path value (B, *l*_*s*_ = 69 vs. A, *l*_*s*_ = 108), in the pathological cornea. As we measure the attenuation of the coherent mean with respect to a specific corneal volume, the degree of transparency may be expressed in terms of photon mean-free path in relation to stromal thickness (*z*_*max*_*/l*_*s*_) or in terms of fraction of the “transmitted” light (*T* = *A*_*out*_/*A*_*in*_ = exp(-*z*_*max*_*/l*_*s*_)). The latter expression may be directly related to Strehl ratio reduction and thus retinal PSF broadening, and as such may be used to create a link with visual function or acuity.

**Table 1:** Scattering mean-free path to quantify the degree of transparency in homogeneous corneal tissues.

	Eye-bank cornea	Pathological cornea
**Mean-free path (*l***_***s***_ **[μm])**	108 ± 2.5	69 ± 2.8
**Stromal thickness (*z***_***max***_ **[μm])**	480	564
**Ratio (*z***_***max***_***/l***_***s***_**)**	4.44	8.17
***T* = *A***_***out***_**/*A***_***in***_ **= exp(-*z***_***max***_***/l***_***s***_**)**	11.8 x 10^−3^	0.28 x 10^−3^

In the last pathological example with visible “hyperreflective” stromal regions (Fig 3C), the residuals of the best fit by a mono-exponential decay are symptomatic of model inadequacy (*b*_*r*_ = 101). The fit by the extended decay model with *z*-dependent *l*_*s*_ confirms that there is a significant deviation from a mono-exponential decay for *z* < 150 μm associated with stromal heterogeneity in that area.

## Conclusion

We described an optical data analysis-based method that allows objective extraction of quantitative measures of corneal transparency from depth-resolved corneal images, addressing the demand for such a means in both the eye-bank and clinical ophthalmology setting.

We demonstrated the feasibility of our approach, in particular of deriving quantitative transparency parameters such as the scattering mean-free path and tissular heterogeneity, from objective analysis of stromal light backscattering with FF-OCT. More specifically, our results on *ex-vivo* corneal specimens demonstrate that computation of the light attenuation (in other words, the attenuation of the coherent mean) in the stroma enables the characterization and standardization of the state of corneal transparency. That is, with heterogeneous, abnormal corneal tissues featuring stromal regions of increased light backscatter, they significantly deviated from the normal exponential stromal-depth profile, as exemplified by Fig 3C. A measure of exponential-decay model inadequacy, such as by computation of the Birge ratio, allowing the estimation of severity of stromal heterogeneity, can thus be used to differentiate heterogeneous from homogeneous tissues. In the latter, homogeneous tissues, the extraction of the photon mean-free path, a major indicator of scattering extent, from the rate of exponential decay allows further quantification of the degree of transparency.

Future studies involving a larger number of corneas, including studies on living corneas from healthy volunteers and patients with corneal disease, will be necessary to determine normative values and thresholds for the scattering mean-free path as well as Birge ratio.

While *in-vivo* development of FF-OCT is underway [23], the proposed analysis may already be implemented in the eye-bank setting [19] and/or into existing clinical depth-resolved corneal imaging methods (e.g., slit-lamp biomicroscopy, OCT, confocal microscopy), enabling objective and quantitative characterization of corneal transparency.

This shall permit, for the first time, standardized staging of corneal disease, providing more accurate markers for diagnosis, progression and monitoring of treatment while enabling effective communication and collaborative research. Specific clinical benefits include earlier diagnosis and facilitating the differentiation of corneal infections (bacterial vs. viral vs. fungal), empowering clinicians to select appropriately targeted drug treatment for the infection in question and decreasing corneal scarring associated with loss of vision. Another example and potential benefit includes the ability to evaluate and refine corneal surgical methods that can directly impact corneal transparency (e.g., refractive surgery, keratoplasty).

In the eye-bank setting, by enabling standardized stromal quality control, the implementation of our method has the potential to improve selection (and rejection) of donor tissues for specific keratoplasty procedures and hence result in decreased graft failure along with better outcomes with respect to visual function and patient satisfaction.

## References

[1] Maurice D. The structure and transparency of the cornea. J Physiol. 1957;136: 263–286. 13429485 10.1113/jphysiol.1957.sp005758 PMC1358888

[2] Plamann K, Aptel F, Arnold CL, Courjaud A, Crotti C, Deloison F, et al Ultrashort pulse laser surgery of the cornea and the sclera. J Opt. 2010;12: 084002.

[3] Whitcher JP, Srinivasan M, Upadhyay MP. Corneal blindness: a global perspective. Bull World Health Organ. 2001;79: 214–221. 11285665 PMC2566379

[4] Garg P, Krishna P, Stratis A, Gopinathan U. The value of corneal transplantation in reducing blindness. Eye. 2005;19: 1106–1114. 10.1038/sj.eye.6701968 16304591

[5] Braunstein RE, Jain S, McCally RL, Stark WJ, Connolly PJ, Azar DT. Objective measurement of corneal light scattering after excimer laser keratectomy. Ophthalmol. 1996;103: 439–443. 10.1016/s0161-6420(96)30674-x 8600420

[6] Fantes FE, Hanna KD, Waring GO III, Pouliquen Y, Thompson KP, Savoldelli M. Wound healing after excimer laser keratomileusis (photorefractive keratectomy) in monkeys. Arch Ophthalmol. 1990;108: 665–675. 10.1001/archopht.1990.01070070051034 2334323

[7] O'Donnell C, Wolffsohn JS. Grading of corneal transparency. Cont Lens Anterior Eye. 2004;27: 161–170. 10.1016/j.clae.2004.08.001 16303539

[8] Lohmann CP, Timberlake GT, Fitzke FW, Gartry DS, Muir MK, Marshall J. Corneal light scattering after excimer laser photorefractive keratectomy: the objective measurements of haze. Refract Corneal Surg. 1992;8: 114–121. 1591203

[9] van de Pol C, Soya K, Hwang DG. Objective assessment of transient corneal haze and its relation to visual performance after photorefractive keratectomy. Am J Ophthalmol. 2001;132: 204–210. 10.1016/s0002-9394(01)01003-0 11476680

[10] Pisella PJ, Auzerie O, Bokobza Y, Debbasch C, Baudouin C. Evaluation of corneal stromal changes in vivo after laser in situ keratomileusis with confocal microscopy. Ophthalmology. 2001;108: 1744–1750. 10.1016/s0161-6420(01)00771-0 11581044

[11] Wang J, Simpson TL, Fonn D. Objective measurements of corneal light-backscatter during corneal swelling, by optical coherence tomography. Invest Ophthalmol Vis Sci. 2004;45: 3493–3498. 10.1167/iovs.04-0096 15452054

[12] Beaurepaire E, Boccara AC, Lebec M, Blanchot L, Saint-Jalmes H. Full-field optical coherence microscopy. Opt Lett. 1998;23: 244–246. 10.1364/ol.23.000244 18084473

[13] Dubois A, Vabre L, Boccara AC, Beaurepaire E. High-resolution full-field optical coherence tomography with a Linnik microscope. Appl Opt. 2002;41: 805–812. 10.1364/ao.41.000805 11993929

[14] Ghouali W, Grieve K, Bellefqih S, Sandali O, Harms F, Laroche L, et al Full-field optical coherence tomography of human donor and pathological corneas. Curr Eye Res. 2015;40: 526–534. 10.3109/02713683.2014.935444 25251769

[15] Huang D, Swanson EA, Lin CP, Schuman JS, Stinson WG, Chang W, et al Optical coherence tomography. Science. 1991;254: 1178–1181. 10.1126/science.1957169 1957169 PMC4638169

[16] Swanson EA, Izatt JA, Hee MR, Huang D, Lin CP, Schuman JS, et al In vivo retinal imaging by optical coherence tomography. Opt Lett. 1993;18: 1864–1866. 10.1364/ol.18.001864 19829430

[17] Izatt JA, Hee MR, Swanson EA, Lin CP, Huang D, Schuman JS. Micrometer-scale resolution imaging of the anterior eye in vivo with optical coherence tomography. Arch Ophthalmol. 1994;112: 1584–1589. 10.1001/archopht.1994.01090240090031 7993214

[18] Stave J, Zinser G, Grummer G, Guthoff R. Modified Heidelberg retinal tomograph HRT. Initial results of in vivo presentation of corneal structures. Ophthalmologe. 2002;99: 276–280. 12058503 10.1007/s003470100535

[19] Irsch K, Grieve K, Borderie M, Ghoubay D, Georgeon C, Borderie V. Full-field Optical Coherence Microscopy for Histology-like Analysis of Stromal Features in Corneal Grafts. J Vis Exp. Forthcoming 2019. 10.3791/57104 36342128

[20] Irsch K, Borderie M, Grieve K, Plamann K, Laroche L, Borderie V. Objective analysis of stromal light backscattering with full-field optical coherence tomographic microscopy shows potential to quantify corneal transparency. In: Proceedings of FiO (Optical Society of America) 2015. paper FW6A.6.

[21] Gelman A, Lee D, Guo J. Stan: a probabilistic programming language for Bayesian inference and optimization. J Educ Behav Stat. 2015;40: 530–543.

[22] Gelman A, Carlin JB, Stern HS, Dunson DB, Vehtari A, Rubin DB. Bayesian Data Analysis. 3rd ed Chapman and Hall/CRC; 2013.

[23] Mazlin V, Xiao P, Dalimier E, Grieve, Irsch K, Sahel JA, et al In vivo high resolution human corneal imaging using full-field optical coherence tomography. Biomed Opt Express. 2018;9: 557–568. 10.1364/BOE.9.000557 29552393 PMC5854058

